# Exploring the Differential Effect of Life Satisfaction on Low and High-Cost Pro-Environmental Behaviors

**DOI:** 10.3390/ijerph19010277

**Published:** 2021-12-27

**Authors:** Salvador del Saz Salazar, Luis Pérez y Pérez

**Affiliations:** 1 Department of Applied Economics II, Universitat de València, Avda. dels Tarongers s/n, 46023 Valencia, Spain; 2 Agrifood Research and Technology Centre of Aragon (CITA), Montañana Avenue, 930, 50059 Zaragoza, Spain; lperez@aragon.es; 3 Agrifood Institute of Aragon (CITA-University of Zaragoza), Montañana Avenue, 930, 50059 Zaragoza, Spain

**Keywords:** subjective well-being, willingness to pay, recycling, public transport emissions, climate change, probit regression

## Abstract

The role of life satisfaction as a determinant of pro-environmental behavior remains largely unexplored in the extant literature. Using a sample of undergraduate students, we explore the effect of life satisfaction on low- and high-cost pro-environmental behaviors. While low-cost pro-environmental behavior has been defined as recycling activities, high-cost pro-environmental behavior is defined in a contingent valuation framework in which respondents are asked about their willingness to pay extra for offsetting CO_2_ emissions, thus avoiding treating the proposed payment as symbolic. Controlling for demographic characteristics and environmental concern, results suggest that life satisfaction has a slightly stronger, and more significant, effect on high-cost pro-environmental behavior than in low-cost pro-environmental behavior. This study also finds that environmental concern and having siblings with a university degree increases the probability of engaging in both behaviors. However, family income is a better predictor of high-cost pro-environmental behavior than of low-cost pro-environmental behavior.

## 1. Introduction

Today, in a context of growing environmental awareness, it is widely recognized that human behavior is responsible for major environmental problems such as global warming and climate change [1,2,3]. In order to address these problems, it is necessary to understand the complex nature of the interactions between human behavior and the environment, i.e., to recognize that some human behaviors can mitigate environmental impact while others exacerbate it [4]. Although over the last years a variety of different terms have been used to describe the array of human behaviors that can reduce environmental impact, in a broad, sense pro-environmental behavior (PEB) can be defined as any “behavior that harms the environment as little as possible, or even benefits the environment” [5] or “any behavior that consciously seeks to minimize the negative impact of one’s actions on the natural and built world” [6].

Over the last forty years, research on PEB has been largely dominated by psychologists covering a wide range of issues given the multi-dimensional structure of PEB [7]. However, integrating insights and models from psychology and economics can result in a better understanding of PEB [8], as it is our intention in this study. Indeed, a growing body of evidence from these fields shows that when humans engage in PEB they experience greater subjective well-being (SWB) or life satisfaction (for a review, see [9]). This positive relationship has been observed both in developed countries [10,11,12,13,14,15,16,17,18] and in developing countries [19,20,21]. In Psychology, SWB is the scientific term for an individual’s evaluation of her experienced positive and negative affect, happiness, or satisfaction with life [22]. Although they are separable constructs, in this article the terms SWB, life satisfaction, and happiness are used interchangeably. Psychologists, in general, use the term happiness with more precision than economists since it is considered to be a narrower concept than SWB, and different from life satisfaction: indeed, happiness and life satisfaction are considered components of SWB [23]. 

In these previous studies, life satisfaction was treated as the dependent variable and modeled as a function of PEB while controlling for socio-demographic characteristics, environmental concern, and other relevant factors. However, it may well be the case that life satisfaction may influence PEB, i.e., there is a causal effect of people’s life satisfaction on their environmental behavior. To the authors’ knowledge, this association remains largely unexplored, since so far only two previous studies have empirically tested the effect of life satisfaction on PEB [24,25]. In both of them, results show that life satisfaction is positively and significantly associated with PEB. Although it is beyond the scope of this research, the possibility of endogeneity cannot be ruled out, i.e., there might be a bidirectional causality between life satisfaction and PEB [25].

This research paper builds on these two previous studies by providing evidence of the impact that life satisfaction has on PEB for a sample of undergraduate students from the University of Valencia, Spain. However, in doing so we go a step further, since we aim to extend the low-cost hypothesis [26] by investigating if life satisfaction—controlling for socio-demographic variables—differentially affects low and high-cost pro-environmental behaviors. The low-cost hypothesis predicts that environmental concern is more likely to be implemented in PEB in situations associated with little inconvenience for the individual than in situations with high costs and great inconvenience. We predict that life satisfaction will have a slightly stronger effect on PEB associated with higher costs than on PEB associated with lower costs, i.e., that individuals that are more satisfied with their lives are more likely to engage in high-cost PEB than those others that are less satisfied with their lives. It is the main aim of this article to present, discuss, and test this hypothesis on the basis of survey data. Based on the understanding that this differential effect of life satisfaction on PEB could be very useful in providing guidance for decision-making, this paper contributes to enrich the literature on the relationship between PEB and life satisfaction.

Although PEB constitutes a diverse and multi-faceted phenomenon [4,27], for the purpose of this article we consider low-cost PEB activities to be recycling glass, paper, and plastic, which demand little effort and time to be undertaken. In parallel, for high-cost PEB we have considered an activity that implies a higher cost for the individual, as is their willingness to pay (WTP) extra per single bus fare in order to offset CO_2_ emissions from public transport that is responsible for climate change. In line with Groot and Steg [28] and Diekmann et al. [26], using public transport instead of a private car is regarded as a high-cost PEB, since it implies giving up many personal advantages such as comfort, independence, convenience, and flexibility. It follows from the above that another contribution of this paper is that, unlike previous studies that ask for “willingness to pay higher taxes” or “willingness to give income to prevent environmental pollution” (see, for example, [24]), in this particular case subjects are confronted with a specific and realistic situation in a Contingent Valuation framework rather than with an abstraction. Indeed, people’s preferences are not measured in the abstract but in terms of concrete items [29]. Therefore, “paying to prevent environmental pollution” is meaningless; what is meaningful is paying higher taxes or prices to prevent a concrete environmental problem in a specific place and in a particular manner, hence the high-cost PEB considered in this research has been clearly and accurately defined in order to avoid respondents treating the payment as symbolic, as could be the case with previous studies. Under a Contingent Valuation framework, individuals are usually asked about their WTP for an improvement in the quality or quantity of a resource (in this case, air quality) that increases their well-being or, less often, their willingness to accept (WTA) in compensation for a well-specified deterioration in the provision of this particular resource [30].

The paper is structured as follows: Section 2 reviews the literature on the relationship between PEB and life satisfaction paying special attention to the role of life satisfaction as a determinant of PEB. Section 3 shows a detailed description of the research design, including the survey, data gathering, and the description of the variables used for the empirical analysis. Section 4 presents the results by distinguishing between low-cost and high-cost PEB. Finally, Section 5 discusses the results and concludes by pointing out that not only attitudes and economic incentives influence PEB, but also how people perceive their lives.

## 2. Literature Review

### 2.1. PEB as a Determinant of Life Satisfaction

Among the plethora of studies that have supported the existence of a positive relationship between PEB and life satisfaction, one of the most comprehensive is the work conducted by Schmitt et al. [18], since the robustness of this relationship was tested using 39 different PEBs. They found that nearly any form of PEB (37 of 39) was positively related to life satisfaction and that this relationship was stronger for behaviors with higher direct costs in time, money, and effort. This study was the first that included perceived ecological threat in this analysis, and they found that while perceived ecological threat reduces life satisfaction, it also motivates PEB, which in turn increases life satisfaction, so the net effect of life satisfaction is practically negligible. Binder and Blankenberg [17] found that the connection between life satisfaction and green lifestyle (or ecologically sustainable behavior) is mostly due to self-image and not due to particular PEB, thus the divergence between self-image and behavior provides a measure of the so-called value–action gap [31]. In a similar vein, Welsch and Kühling [32] found a significantly positive relationship between green self-image and life satisfaction in a pool of 35 European countries, and their results suggest that the well-being benefit from green self-image derives from conformity to a social norm, i.e., as individuals conceptualize green self-image as a social norm, then acting in accordance with what is socially considered as appropriate enhances their well-being [33]. Another finding is that pro-environmental consumption choices cannot be consistent with utility maximization because individuals do not hold perfect information about the benefits and costs of their decisions [13,14].

In a study conducted in Sweden by Kaida and Kaida [15], as respondents were asked to rate their state of life satisfaction both in the present and in the future, it was found that PEB not only enhances present life satisfaction, but also expectations of better future life satisfaction. However, the most interesting finding of this study was that expectations of better future life satisfaction are negatively and significantly associated with PEB in the present. A possible interpretation of this negative relationship is that people who expect a happier life in the future have less incentive to engage in PEB in the present because it could be very costly to them. 

Finally, an exception to the positive association between PEB and life satisfaction can be found in a recent work by Binder et al. [34]. In fact, using a sample of undergraduate students from the University of Granada, Spain, they found that higher levels of PEB are associated with lower life satisfaction. According to the authors, this result is probably due to the low disposable income of Spanish students. In contrast, they found that green self-image is positively related to life satisfaction irrespective of green behavior.

### 2.2. Life Satisfaction as a Determinant of PEB: The Flip Side of the Coin

As previously said, little attention has been paid to the role of life satisfaction as a determinant of PEB and environmental concern. The study by Sulemana [24] is the first attempt to shed light on this issue. Indeed, based on two questions from the World Values Survey, he defines environmental concern as “willingness to give income” and “willingness to pay higher taxes” to prevent environmental pollution. Then, using ordered probit regression models, he explains these two dependent variables as a function of happiness and other control variables (demographic characteristics, economic circumstances, perceived environmental quality, etc.) for a sample of 7 Sub-Saharan countries and 11 developed countries. He concludes that happiness is an important determinant of environmental concern for both samples, although the effect is slightly higher for African respondents than for their counterparts in developed countries. More specifically, it is found that a unit increase in happiness raises the proportion of respondents’ “willing to give income” to prevent environmental pollution by about 2.3% for the African countries and about 1.6% for the advanced countries. This difference is even greater in the case of “willingness to pay higher taxes”.

Wang and Kang [25], using data from the China General Social Survey, found that life satisfaction has an important effect on the probability of participating both in private and public PEBs as saving water and energy, reducing the use of environmentally harmful products, paying higher environmental taxes, and reducing living standards in order to safeguard the environment. Their results also suggest that environmental concern can be regarded as an important channel through which people’s life satisfaction can play a crucial role in promoting their PEB.

Finally, Casaló et al. [35], in a study aimed at analyzing the influence of environmental attitudes and environmental knowledge on four PEBs (separating the trash, using street trash cans, using energy-efficient light bulbs, and using recycling centers), included life satisfaction in the set of covariates and found that it was positively associated with all the PEBs. Nevertheless, apart from that, they did not investigate further the relationship between life satisfaction and PEB, as was the case with the other two above-mentioned studies. 

## 3. Materials and Methods

### 3.1. Procedure and Participants

The present analysis is based on a survey conducted in spring 2018 amongst undergraduate students of the University of Valencia, Spain. Using the free-survey tool Google Docs, a link to the questionnaire was emailed to 509 students from different majors who answered it on their personal computer, tablet, or cell phone. Respondents were informed about the anonymity and confidentiality of the study and about their right to withdraw. Participation was voluntary and no incentives were provided. Answers were collected automatically on an Excel compatible spreadsheet, which makes Google Docs a convenience and time-saving survey-creation tool. Participation in the survey was voluntary and no incentives were provided to the respondents. Two weeks later, a reminder email was sent to the non-respondents. The number of completed surveys received was 450, amounting to a response rate of 88.4%. Although lower than the response rate obtained by Binder et al. [34] in a similar study also carried out in Spain, this is a fairly high response rate considering that, in general, web surveys yield lower response rates than other survey modes [36].

### 3.2. Dependent Variables

As previously mentioned, we have divided the assessment of individual PEB in two categories: low-cost and high-cost PEB. The former was captured by asking the respondents how often they engage in the following behaviors using a 5-point Likert scale (1 = “never”; 2 = “rarely”; 3 = “sometimes”; 4 = “very often”; 5 = “always”): “recycling glass”, “recycling paper”, and “recycling plastic”. Self-reports of engagement in PEB are commonly used to reflect objective PEB given their advantages (low cost, ease of use, and flexibility). However, inaccuracies may arise since they are subjective in nature, and because sometimes individuals tend to over-report their PEB due to the existence of a social desirability bias [37].

As the recycling questions (low-cost PEB) are categorical and ordered, the appropriate regression model to explain these behaviors is the ordered probit model [38,39]. Indeed, probit regressions are amongst the most frequently used methods to find out the factors that influence on PEB [40]. In this model, the latent variable $LCPEB_{i}^{*}$ represents the true level of individual *i*’s (low-cost) pro-environmental behavior, and it is a function of observable characteristics (life satisfaction and other control variables) and an error term:

(1) $$LCPEB_{i}^{*}=\beta ls_{i}+\gamma x_{i}+u_{i}\;$$

where *ls_i_* is the life satisfaction reported by the respondent *i*, *x_i_* is a vector of the observable characteristics of this individual as age, gender, income, degree of environmental concern, etc., *β* and δ are parameters to be estimated, and *u_i_* is an error term assumed to be normally distributed across observations. As usual, $LCPEB_{i}^{*}$ is unobserved. What the research does observe is the ordinal category in which the respondent’s behavior is reported. As these are recycling questions, the researcher observes the reported frequency *F_i_*, where *F_i_* depends on the true level of green behavior [41]:

(2) $$F_{i}=\left\{\begin{array}{c} AL\;\mathrm{if}\;LCPEB_{i}^{*}>k_{4}\;\\ VO\;\mathrm{if}\;k_{4}\geq LCPEB_{i}^{*}>k_{3}\\ SO\;\mathrm{if}\;k_{3}\geq LCPEB_{i}^{*}>k_{2}\\ RA\;\mathrm{if}\;k_{2}\geq LCPEB_{i}^{*}>k_{1}\\ NE\;\mathrm{if}\;k_{1}\geq LCPEB_{i}^{*} \end{array}\right\}$$

where the *k’s* are the cut points that determine the intervals of the frequency scale, AL is “always”, VO is “very often”, SO is “sometimes”, RA is “rarely”, and NE is “never”. Thus, the probability that individual *i* reports a recycling frequency of “sometimes (SO)” is:

(3) $$\begin{array}{c} Prob\left(F_{i}=SO\right)\;=Prob\left(k_{3}\geq \beta ls_{i}+\gamma x_{i}+u_{i}>k_{2}\right)\\ \;\;\;\;\;\;=\Phi \left(k_{3}-\beta ls_{i}+\gamma x_{i}+u_{i}\right)-\left(k_{2}-\beta ls_{i}+\gamma x_{i}+u_{i}\right) \end{array}$$

where $\Phi \left(\cdot \right)$ is the standard normal c.d.f.

The question related to the high-cost PEB asked respondents for their WTP in order to offset CO_2_ emissions from public transport that are responsible for climate change. Rather than being an abstraction, this question was framed in a contingent valuation scenario that allows to put a value on environmental goods that are not usually traded in the marketplace [42], thus making credible the proposed trade-off between respondent’s WTP and the change in environmental quality proposed [43]. However, in previous studies on the relationship between life satisfaction and PEB, the object of valuation was characterized in rather general terms since respondents were asked for “willingness to pay higher taxes” or “willingness to give income” to prevent “environmental pollution”. This can be problematic because the vaguer and less specific the commodity and payment mechanism, the more likely respondents are to treat the valuation as symbolic [29]. Indeed, “paying for protecting the environment” is meaningless. Respondents should be confronted with something concrete and there should be a real sense of commitment, otherwise answers can be answered on the fly. 

More specifically, in this study respondents were presented with the following contingent valuation framework:

*“Imagine that there was a program aimed at substituting the current diesel bus fleet in the city public transport system for hybrid electric buses. These new buses would be more efficient and accordingly would emit less greenhouse gases than diesel buses. Nevertheless, they would be between 40% and 70% more costly. Considering the environmental benefits of these buses and that this program will be implemented only if a majority of respondents are in favor of the program, would you willing to pay higher bus fares to support this program?”*

In this case, there are two possible outcomes: accepting (*y_i_* = 1) or rejecting (*y_i_* = 0) the proposed payment. Therefore, an appropriate regression model to explain this binary outcome is the probit model. Following Cameron and Trivedi [38], the general formulation for a probit model is:

(4) $$Prob\;(y_{i}=1|\;ls_{i}\;,x_{i})=\Phi \;\left(\alpha +\beta ls_{i}+\gamma x_{i}+u_{i}\right)$$

where *ls_i_* is the life satisfaction reported by the respondent *i*, *x_i_* is a vector of the observable characteristics of this individual as age, gender, income, degree of environmental concern, etc., *β* and δ are parameters to be estimated, *u_i_* is an error term assumed to be normally distributed across observations, and $\Phi \left(\cdot \right)$ is the cumulative distribution function for the standard normal.

### 3.3. Explanatory Variables

Subjective well-being, operationalized through a “satisfaction with life” construct, was measured using the following question:

*All things considered, how satisfied are you with your life as a whole these days? Please, choose a value in the eleven-point scale where “0” means “completely dissatisfied” and “10” means “completely satisfied”*.

The evidence seems to favor the use of an 11-point scale (0–10) for single-item measures of life evaluation, since this scale has significant advantages in terms of data quality over shorter scales apart from being a valid and reliable indicator of well-being [44,45]. In this particular case, besides its desirable properties, this 11-point scale was chosen because in Spain undergraduate students are very familiar with this scale, since it is used as a grading scale at the university [46]. In order to avoid question order effects, the life-satisfaction question was not only placed on the first section of the questionnaire, but also before other sensitive survey questions [47,48].

With respect to explanatory variables, respondents were asked to answer several statements intended to measure environmental concern following Diekmann and Preisendörfer [26]. These statements are related to cognitive, conative, and affective aspects of the individual with regard to the environment, e.g., respondent’s agreement with the sentence “I am afraid when I think about environmental conditions for future generations”. These statements, measured on a 5-point Likert scale (1 = “strongly disagree”; 5 = “strongly agree”), and their descriptive results are shown in Table 1. These questions were included in the questionnaire because a number of studies document that environmental concern may translate into environmental action (see, among others, [49,50,51]), although often this translation is not as straightforward as it seems due to the existence of a value–action gap [6,52,53]. It can be seen from the table that overall respondents exhibit a high degree of environmental concern since, for example, 92% of them agree or strongly agree with the statement that “the conservation and protection of the environment is crucial”, 85% of them are worried about the environmental conditions for the future generations, and 88% of them believe that climate change is real. However, when protecting the environment is at the cost of jobs, the percentage of those that agree or strongly agree is much lower (45%). This is an expected result considering that Spain has one of the highest unemployment rates in the European Union.

Following existing research on PEB, subjective well-being, and contingent valuation, we also included a set of explanatory variables covering personal and socio-demographic information about the respondents. These variables and others are described in Table 2. As PEB is expected to be correlated with income, respondents were asked about household monthly income. In particular, respondents indicated their household income by selecting one of the eleven €300 intervals ranging from interval 1 (€0–€300) to interval 11 (>€3900). Next, household income was calculated as the midpoint of the selected income bracket divided by the number of members in the household. The average income was €526.9.

## 4. Results

### 4.1. Life Satisfaction

**Figure 1:**
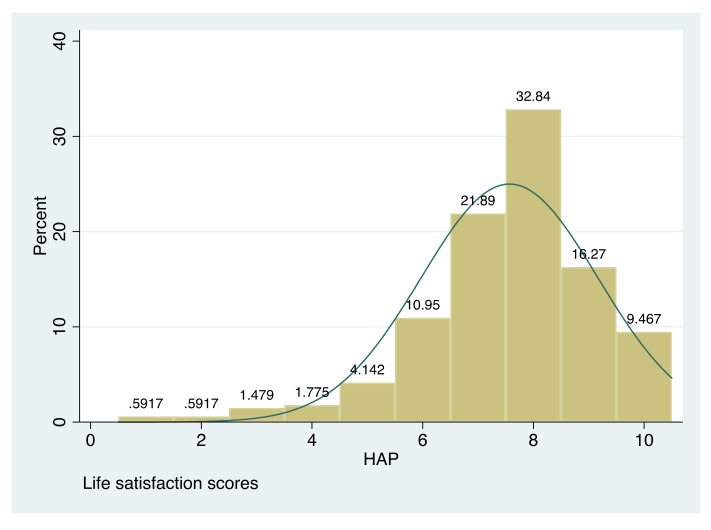
Life satisfaction scores.

Figure 1 shows the distribution of life satisfaction scores in our sample, being the average life satisfaction reported 7.57, a value that is higher than that for Spain: 7.3 and 6.7 according to OECD [54] and Veenhoven [55], respectively. It is also higher than the average life satisfaction reported by undergraduate students (7.06) in the study conducted by Binder et al. [34].

### 4.2. Low-Cost PEB

Table 3 summarizes the responses to the three recycling questions. Overall, our data show that for the three behaviors the level of engagement is quite high, i.e., more than 65% of participants answered “very often” or “always” when asked about their recycling behaviors. 

The coefficients of the estimated models for each recycling behavior are shown in Table 4. As expected, the regression results show that life satisfaction is positively and significantly correlated with the three low-cost PEB considered (at the 10% level for recycling glass and plastic, and at the 5% level for recycling paper). These results are in line with those of Casaló et al. [35], Wang and Kang [25], and Sulemana [24]. Another interesting result is that the level of income, adjusted by the number of family members, only affects the frequency of engagement in recycling glass, while for the other two recycling behaviors this variable is not statistically significant. This result seems to contradict the affluence hypothesis that suggests that affluent individuals have a higher probability of engaging in PEB [56,57]. However, affluence is not necessarily a prerequisite for engaging in PEB, especially in the case of low-cost PEBs such as recycling activities. Indeed, empirical evidence suggests that low-cost behaviors are more strongly related to altruistic and biospheric values than high-cost behaviors, since PEB is often seen as acting morally right [58]. Spain has a well-developed system for recycling glass, paper, and plastic. Recycle bins can be found all over the country, so these recycling activities demand little effort and time to be undertaken. Unlike other European countries, such as Finland or Demark, market-based policies aimed at increasing recycling rates, such as deposit–refund programs, have not yet been implemented in Spain. Other PEBs are highly constrained by income since they are costly to perform although they confer higher social status [59], e.g., reinsulating homes and buying a hybrid or electric car. Interestingly, having a sibling that currently is in college or that has completed their degree increases the probability of engaging in recycling activities, being this positive relationship is statistically significant at the 1% level for the three recycling activities.

Among the different statements intended to capture environmental concern, the only ones that are positively and significantly correlated with the three recycling activities are “environmental protection measures should be implemented even if this reduces the number of jobs in the economy” and “the conservation and protection of the environment is crucial”. In the case of the first statement, this relationship is significant at the 1% level for each one of the three recycling behaviors, while for the second statement it is significant at the 5% level for recycling glass and plastic and only at the 10% level for recycling paper. Other statements, such as “climate change is real since average temperatures have increased and climate catastrophes are becoming more common” and “most citizens do not act in a responsible way with regard to the environment”, increase the probability of engaging in recycling glass and plastic. Additionally, the results also show that students who on their daily commutes choose motorized transport modes (car, motorbike, bus, or metro), instead of more environmentally friendly non-motorized transport modes, are less likely to recycle glass and paper.

While in linear models the estimated coefficients have a direct interpretation, since they represent the estimated change in the value of the dependent variable associated with a unit increase in the corresponding independent variable (marginal effect), the same does not hold for ordered probit models. Indeed, the nonlinearity of these models implies that the relationship between a change in the value of an independent variable and the estimated change in the probability of an outcome cannot be directly determined from the variable’s coefficient. Rather, this magnitude varies depending on the extent of the change in the independent variable of interest, the starting value of this same variable, and the values of the other explanatory variables in the model [60]. Thus, for each recycling behavior, marginal effects have been calculated in order to know how much the probability of the outcome variable changes when the value of an explanatory variable is changed, holding all other variables constant at their mean values. Therefore, Table 5, Table 6 and Table 7 report marginal effects for “never”, “rarely”, “sometimes”, “very often”, and “always”, since the marginal effect will be different for each frequency level, *F_i_*, of the three recycling behaviors. Focusing first on life satisfaction, a unit increase in this variable increases the probability of an individual reporting “always” by 2.4, 2.9, and 2.3% for recycling glass, paper, and plastic, respectively. Thus, the results suggest an important influence of life satisfaction on the three recycling behaviors. 

Students that have a sibling currently in college are approximately 12% more likely to report “always” for recycling glass than the rest of students, while for recycling paper and plastic these percentages are, respectively, 16 and 11 per cent. On the other hand, as recycling activities can be considered as low-cost PEB, the effect of household income-adjusted by the number of family members on the probability of reporting “always” is only statistically significant for recycling glass and almost negligible, i.e., a unit change in income increases this probability by only 0.02%.

Variables related to environmental concern have an important average marginal effect. Indeed, a discrete change in the variable JOBS (“environmental protection measures should be implemented even if this reduces the number of jobs in the economy”) increases this probability by 9.2, 6.8, and 6.8% for recycling plastic, glass, and paper, respectively. This is an interesting result since, in some way, it shows to what extent highly-environmentally-concerned individuals are willing to protect the environment even at the expense of jobs. The variable CLIMA (“climate change is real since average temperatures have increased and climate catastrophes are becoming more common”) is only statistically significant in explaining “recycling paper”, so a discrete change in this variable increases the probability of an individual reporting “always” by 8%. However, the largest effect on reporting “always” is due to a discrete change in the variable CRUCIAL (“the conservation and protection of the environment is crucial”) since it increases this probability by over 60% for recycling glass and plastic, and by over 30% for recycling paper. On the other hand, a discrete change in the variable NRESP (“most citizens do not act in a responsible way with regard to the environment”) increases the probability of reporting “always” by 10 and 8% for recycling glass and plastic, respectively, while in the case of recycling paper it is not statistically significant. Finally, choosing a motorized transport mode when commuting reduces the probability that an individual “always” recycles glass by 9% and increases the probability of reporting “never” by 2.9%, while in the case of recycling paper the changes in the probabilities are, respectively, −7.7 and 2.2%. 

### 4.3. High-Cost PEB

As previously mentioned, in a contingent valuation framework, respondents were asked whether they were willing to pay extra bus fares in order to offset CO_2_ emissions from public transport that are responsible for climate change. In this case, there are two possible outcomes: accepting (*y_i_* = 1) or rejecting (*y_i_* = 0) the proposed payment. Therefore, an appropriate regression model to explain this binary outcome is the probit model. However, the non-linearity of this model implies that, other than sign and significance, the coefficients do not reflect marginal impacts on the probability of accepting the proposed payment [61]. Accordingly, marginal effects are calculated for each variable by allowing one independent variable to change while keeping the rest of independent variables at their respective means. 

Results show that, overall, respondents support the adoption of this alternative technology since 67% of them were willing to pay extra per single bus fare. In a previous study, Lin and Tan [62] found that in the four most developed Chinese cities, about 80% of the respondents were willing to pay extra to support the adoption of this hybrid technology. As expected, there is a positive and significant effect of life satisfaction on agreeing to pay extra per single bus ride in order to offset CO_2_ emissions from public transportation (see Table 8). In particular, a unit increase in life satisfaction increases the proportion of respondents willing to pay by 3.2%. This effect is slightly stronger than the effect for the three recycling behaviors. This finding is in line with the results of Sulemana [24] and Wang and Kang [25], which also reported a positive correlation between life satisfaction and willingness to pay to protect the environment.

In a contingent valuation scenario, a crucial variable to test its theoretical validity is income. Past empirical evidence and economic theory suggest that income has a positive effect on WTP estimates [63]. Regression results show that probability of accepting the proposed payment is positively and significantly (at 10% level) related to household income, adjusted by the number of family members. A unit increase in household income raises the probability of being willing to pay by 0.01%. This positive relationship between these two variables was also found in previous research on willingness to pay for alternative energy buses [62,64]. 

Interestingly, having a sibling with a university degree increases the probability of being willing to pay by 7.6%, while, as expected, those respondents that answered “No, totally sure” to the question “Would you use more public transport if the city’s bus fleet were renewed and become entirely hybrid?” (variable PTRANS) are approximately 20% less willing to pay than the rest, this being, by fare, the highest marginal effect among all the explanatory variables.

Regarding the set of variables related to environmental concern, on average respondents who were “highly environmentally concerned” (variable HECONC), i.e., those that answered “agree” or “strongly agree” in each one of the different “statements for measuring environmental concern” shown in Table 1, are 12.6% more likely to accept the proposed payment. As in the previous model concerning recycling behaviors, this result confirms the positive relationship between environmental concern and PEB. 

## 5. Discussion

The low-cost hypothesis [26] predicts higher correlations between environmental concern and PEB in situations and circumstances characterized by low cost and little inconvenience for the individual, i.e., the smaller the perceived cost of engaging in a behavior, the greater the likelihood that attitudes are transformed into behavior. However, in this study we show that not only attitudes and economic incentives influence PEB, but also how people perceive their lives. On the basis of mainly self-reported behavior we extend the low-cost hypothesis, demonstrating that life satisfaction has a significant influence on PEB. While this is not a new finding, since the causal relationship between these two variables has been recently shown by Sulemana [24] and Wang and Kang [25], what is new is that our results indicate that life satisfaction differentially affects low-cost and high-cost PEB, thus contributing to the literature on PEB. Indeed, it seems that life satisfaction has a slightly stronger (and more significant) effect on high-cost PEB than in low-cost PEB, i.e., happier individuals are more likely to engage in high-cost PEB than those others that are less satisfied with their lives. Admitting that there could be a bidirectional causality between PEB and life satisfaction, as suggested by Sulemana [24], and engaging in high-cost PEB assessed in the currency of life satisfaction might not be so costly to the individual who performs this behavior, since the increase in their life satisfaction would be slightly higher than when they engage in low-cost PEB. In any case, these returns of PEB in terms of life satisfaction would need further research.

Overall, our results do not fully confirm the low-cost hypothesis since it seems that environmental attitudes predict both low-cost and high-cost PEB. For example, the variables “the conservation and protection of the environment is crucial” and “environmental protection measures should be implemented even in this reduces the number of jobs in the economy” are significant predictors of the three low-cost recycling behaviors, while in the case of high-cost PEB, the fact of being “highly environmentally concerned” and “saving water at home” have an important marginal effect on the probability of accepting to pay higher bus fares in order to reduce the emissions from urban transport.

## 6. Conclusions

This research, using data from a survey of undergraduate students, has empirically investigated the relationship between life satisfaction and low-cost and high-cost pro-environmental behaviors. The results show a positive and significant effect of life satisfaction on both types of pro-environmental behavior, although this effect is stronger and more statistically significant for the high-cost pro-environmental behavior which has been defined in a contingent valuation framework. Considering the paucity of studies that have so far investigated the role of life satisfaction as a determinant of pro-environmental behavior, there is the need for more studies to further investigate and to throw more light on the exact nature of the relationship between these two variables.

Finally, there are at least two limitations of our study that should be considered when interpreting its results. Firstly, although student samples are not a rare occurrence in the literature on PEB since they are convenient, cheap, and readily available (see [19,34,41,65,66], among others), the fact of having used a sample of undergraduate students means that the findings from the analysis carried out cannot be directly extrapolated to the general population. Consequently, building on the findings presented here, the next logical step would be to conduct a survey for the general population paying special attention to its representativeness, which would allow more meaningful results to be obtained. Secondly, as in many previous studies, our results are based on self-reports on PEB considering its advantages, such as low cost and relative ease of use, and resting on the assumption that they accurately reflect the individuals’ actual behavior. However, empirical evidence in this respect is mixed. Indeed, while some studies suggest that individuals tend to over-report their PEB as a consequence of social desirability concerns [67,68,69], and the wish to live more sustainable or to show their green credentials, thus making their answers more about self-image [50,70], others point out that self-reports are valid, convenient, and cost-effective indicators of behavior [37], and that social desirability bias does not have a strong effect on the way people respond to questions addressing environmental behaviors [71]. 

## Figures and Tables

**Table 1:** Statements for measuring environmental concern and their descriptive statistics following Diekmann and Preisendörfer [26].

Statement	Mean	Std. dev.	++
I am afraid when I think about environmental conditions for future generations	4.32	0.83	85.6
If we continue our current style of life, we are approaching an environmental catastrophe	4.37	0.87	86.5
Watching TV or reading in the newspapers about environmental problems, I am often embarrassed and angry	4.11	0.96	77.3
The great majority of people do no act in an environmentally responsible way	4.32	0.78	85.3
There are limits of economic growth that the industrialized world has reached or will reach very soon	3.82	1.09	64.9
In my opinion, environmental problems are greatly exaggerated by proponents of the environmental movement *	1.90	0.95	8.0
It is still true that politicians do much too little to protect the environment	4.24	0.95	84.7
To protect the environment, we all should be willing to reduce our current standard of living	3.69	1.16	60.8
Environmental protection measures should be implemented even in this reduces the number of jobs in the economy	3.34	1.03	45.5
Climate change is real since average temperatures have increased and climate catastrophes are becoming more common (floods, prolonged droughts, hurricanes, etc.). **	4.39	0.79	88.5
The conservation and protection of the environment is crucial **	4.52	0.67	92.2

Note: ++ means percentage of respondents that answered “agree” or “strongly agree” on the 5 point Likert scale. * in this case disagreement is seen as indicating higher environmental concern. ** These two additional statements were proposed by the authors.

**Table 2:** Explanatory variables.

Variable	Definition	Mean	S.D.	% of 1 s
LSATIS	Self-reported life satisfaction using an 11-point scale where “0” means “completely dissatisfied” and “10” means “completely satisfied”.	7.57	1.59	
INCOME	Respondent’s household monthly income in per capita terms (€).	526.9	271.7	
SIBLING	Indicates the number of brothers with university studies that the respondent has.	0.51	0.67	
TRANSM	1 if the transport mode usually chosen by the respondent is a motorized transport, 0 otherwise (non-motorized).			72.09
PTRANS	1 if the respondent on a 5-point scale (1 = “No, totally sure”; 5 = “yes, totally sure”) answered “No, totally sure” to the question: Would you use more public transport if the Valencia’s bus fleet were renewed and become entirely hybrid? 0 otherwise.			6.92
JOBS	Respondent’s agreement with the sentence “Environmental protection measures should be implemented even in this reduces the number of jobs in the economy” on a 5-point scale (1 = strongly disagree; 5 = strongly agree)	3.34	1.03	
CLIMA	Respondent’s agreement with the sentence “Climate change is real since average temperatures have increased and climate catastrophes are becoming more common” on a 5-point scale (1 = Strongly disagree; 5 = Strongly agree)	4.39	0.79	
ENVCRU	1 if the respondent on a 5-point scale (1 = “Strongly disagree”; 5 = “Strongly agree”) answered “agree” or “strongly agree” to the sentence “The conservation and protection of the environment is crucial, 0 otherwise.			92.3
NRESP	1 if the respondent on a 5-point scale (1 = “Strongly disagree”; 5 = “Strongly agree”) answered “agree” or “strongly agree” to the sentence “Most citizens do not act in a responsible way with regard to the environment”.			85.4
SWATER	1 if the respondents on a 5-point scale (1 = ”Never”; 5 = “Always”) answered “frequently” or “always” to the question “Do you usually carry out activities aimed at saving water in your home?”, = otherwise.			90
HECONC	1 if the respondent is highly environmentally concerned, 0 otherwise. Highly environmentally concerned in this case means that the respondents answered “agree” or “strongly agree” when asked about the different “statements for measuring environmental concern” shown in Table 2.			17.7

**Table 3:** Recycling behaviors.

Category	Glass Recycling		Paper Recycling		Plastic Recycling	
	Freq.	%	Freq.	%	Freq.	%
Never	25	5.61	18	4.00	25	6.01
Rarely	40	8.97	49	10.89	51	11.36
Sometimes	58	13.00	89	19.78	75	16.70
Very often	103	23.09	141	31.33	125	27.84
Always	220	49.33	153	34.00	171	38.08
N	446	100.00	450	100.00	449	100.00

**Table 4:** Estimated parameters for recycling behaviors (low-cost PEB): ordered probit regressions.

	Recycle Glass		Recycle Paper		Recycle Plastic	
Variable	Coeff.	Z val.	Coeff.	Z val.	Coeff.	Z val.
LSATIS	0.0675 *	1.72	0.0867 **	2.31	0.0666 *	1.76
INCOME	0.0006 ***	2.61	0.0001	0.80	0.0002	1.24
SIBLING	0.3414 ***	2.63	0.4874 ***	3.92	0.3291 ***	2.64
JOBS	0.1893 ***	3.05	0.2039 ***	3.45	0.2634 ***	4.41
CLIMA	0.2314 ***	2.77	0.0402	0.51	0.0283	0.36
ENVCRU	1.8113 **	2.48	1.0329 *	1.67	1.8093 **	2.51
NRESP	0.2821 **	2.05	0.1870	1.45	0.2537 *	1.94
TRANSM	−0.2557 *	1.77	−0.2302 *	−1.69	−0.1427	−1.04
Log L LR χ^2^ (8) Prob > χ^2^ Pseudo R^2^ N	−410.28 55.25 0.000 0.063 387		−441.84 46.01 0.000 0.049 387		−443.33 47.46 0.000 0.051 387	

Note: *** Significant at 1% level; ** significant at 5% level; * significant at 10% level.

**Table 5:** Average marginal effects for recycling glass (low-cost PEB).

Variable	NE	Z val.	RA	Z val.	SO	Z val.	VO	Z val.	AL	Z val.
LSATIS	−0.0078 *	−1.66	−0.0075 *	−1.69	−0.0057 *	−1.69	−0.0031	−1.57	0.0243 *	1.74
INCOME	−0.0000 **	−2.41	−0.0000 **	−2.49	−0.0000 **	−2.52	−0.0000 **	−2.23	0.0002 ***	2.68
SIBLING	−0.0395 **	−2.43	−0.0383 **	−2.51	−0.0289 **	−2.53	−0.0161 **	−2.27	0.1228 ***	2.70
TRANSM	0.0296 *	1.70	0.0286 *	1.73	0.0216 *	1.73	0.0120	1.64	−0.0920 *	−1.79
JOBS	−0.0219 ***	−2.74	−0.0212 ***	−2.86	−0.0160 ***	−2.92	−0.0089 **	−2.46	0.0681 ***	3.15
CLIMA	−0.0267 ***	−2.56	−0.0259 ***	−2.64	−0.0195 ***	−2.63	−0.0109 **	−2.27	0.0832 ***	2.84
ENVCRU	−0.2096 **	−2.44	−0.2031 **	−2.34	−0.1532 **	−2.27	−0.0855 **	−1.98	0.6516 **	2.49
NRESP	−0.0326 **	−1.96	−0.0316**	−1.99	−0.0238 **	−2.01	−0.0133 *	−1.87	0.1015 **	2.09

Note: *** Significant at 1% level; ** significant at 5% level; * significant at 10% level. AL is “always”, VO is “very often”, SO is “sometimes”, RA is “rarely”, and NE is “never”.

**Table 6:** Average marginal effects for recycling paper (low-cost PEB).

Variable	NE	Z val.	RA	Z val.	SO	Z val.	VO	Z val.	AL	Z val.
LSATIS	−0.0082 **	−2.11	−0.0117 **	−2.26	−0.0100 **	−2.26	−0.0009	−0.65	0.0290 **	2.33
INCOME	−0.0000	−0.79	−0.0000	−0.80	−0.0000	−0.80	0.0000	0.51	0.0000	0.88
SIBLING	−0.0462 ***	−3.11	−0.0657 ***	−3.61	−0.0564 ***	−3.80	0.0051	0.65	0.1632 ***	4.10
TRANSM	0.0218	1.62	0.0310 *	1.66	0.0266 *	1.67	−0.0024	−0.61	−0.0771 *	−1.71
JOBS	−0.0193 ***	−2.86	−0.0275 ***	−3.25	−0.0235 ***	−3.36	−0.0021	0.66	0.0682 ***	3.55
CLIMA	−0.0038	−0.50	−0.0054	−0.51	−0.0046	−0.51	0.0004	0.40	0.0134	0.51
ENVCRU	−0.0979	−1.61	−0.1393 *	−1.65	−0.1195	−1.61	0.0108	0.64	0.3459 *	1.66
NRESP	−0.0177	−1.39	−0.0252	−1.42	−0.02164	−1.45	−0.0019	0.60	0.0626	1.46

Note: *** Significant at 1% level; ** significant at 5% level; * significant at 10% level. AL is “always”, VO is “very often”, SO is “sometimes”, RA is “rarely”, and NE is “never”.

**Table 7:** Average marginal effects for recycling plastic (low-cost PEB).

Variable	NE	Z val.	RA	Z val.	SO	Z val.	VO	Z val.	AL	Z val.
LSATIS	−0.0082 *	−1.70	−0.0080 *	−1.74	−0.0060 *	−1.72	−0.0007	−0.67	0.0231 *	1.77
INCOME	−0.0000	−1.22	−0.0000	−1.23	−0.0000	−1.23	−0.0000	−0.63	0.0000	1.24
SIBLING	−0.0408 **	−2.45	−0.0397 **	−2.54	−0.0300 **	−2.56	0.0037	0.72	0.1144 ***	2.70
TRANSM	0.0177	1.02	0.0172	1.04	0.0130	1.03	0.0016	0.60	−0.0496	−1.04
JOBS	−0.0327 ***	−3.66	−0.0318 ***	−4.00	−0.0240 ***	−4.08	−0.0030	−0.72	0.0916 ***	4.65
CLIMA	−0.0035	−0.35	−0.0034	−0.35	−0.0025	−0.36	−0.0003	−0.32	0.0098	0.36
ENVCRU	−0.2246 **	−2.47	−0.2185 **	−2.37	−0.1654 **	−2.29	−0.0206	−0.69	0.6292 **	2.51
NRESP	−0.0315 *	−1.89	−0.0306 *	−1.89	−0.0231 *	−1.91	−0.0028	−0.69	0.0882 *	1.96

Note: *** Significant at 1% level; ** significant at 5% level; * significant at 10% level. AL is “always”, VO is “very often”, SO is “sometimes”, RA is “rarely”, and NE is “never”.

**Table 8:** Estimated parameters for high-cost PEB: probit regression.

Variable	Coefficient	Z val.	Marg. Effects	Z val.
LSATIS	0.0927 **	1.99	0.0320 **	2.02
INCOME	0.0004 *	1.77	0.0001 *	1.79
SIBLING	0.2209 *	1.94	0.0764 **	1.97
PTRANS	−0.5917 **	−2.11	−0.2046 **	−2.15
SWATER	0.5372	2.27	0.1858 **	2.33
HECONC	0.3647 *	1.71	0.1261 *	1.74
Log L LR χ^2^ (6) Prob > χ^2^ Pseudo R^2^ N	−197.30 25.30 0.0003 0.060 387			

Note: ** significant at 5% level; * significant at 10% level.

## Data Availability

Survey data available on request to the corresponding author.
